# Validation of the extended Nordic Musculoskeletal Questionnaire among healthcare professionals in Singapore

**DOI:** 10.3389/fpubh.2026.1840201

**Published:** 2026-05-19

**Authors:** Pamela E-Wei Gopal, Gwendoline Wan Hua Tan, Yu Heng Kwan, Jie Kie Phang, Lian Leng Low

**Affiliations:** 1 Department of Post-Acute and Continuing Care, SingHealth Community Hospitals, Singapore, Singapore; 2 SingHealth Duke-NUS Family Medicine Academic Clinical Program, Singapore, Singapore; 3 Program in Health Services and Systems Research, Duke-NUS Medical School, Singapore, Singapore; 4 Department of Rheumatology and Immunology, Singapore General Hospital, Singapore, Singapore; 5 SingHealth Duke-NUS Medicine Academic Clinical Program, Singapore, Singapore; 6 Centre for Population Health Research and Implementation, SingHealth Office of Regional Health, Singapore, Singapore; 7 Research and Translation Innovation Office, SingHealth Community Hospitals, Singapore, Singapore; 8 Division of Population Health and Integrated Care, Singapore General Hospital, Singapore, Singapore

**Keywords:** extended Nordic Musculoskeletal Questionnaire, healthcare workers (HCWs), psychometric validation, Singapore, work-related musculoskeletal disorders, validity, reliability

## Abstract

**Background:**

Work-related musculoskeletal disorders are highly prevalent among healthcare professionals and contribute to disability, reduced productivity and compromised patient care delivery. The Extended Nordic Musculoskeletal Questionnaire is a comprehensive tool used for gathering data on musculoskeletal symptoms and their impact, however its measurement properties have not been validated in Singapore. This study aimed to evaluate the reliability and validity of the extended Nordic Musculoskeletal Questionnaire in measuring musculoskeletal disorders (MSDs) among healthcare professionals in two community hospitals in Singapore.

**Methods:**

A total of 251 nurses and therapists working in two community hospitals were recruited in a cross-sectional study conducted between March 2024 and August 2024, following the Consensus-based Standards for selection of Health Measurement Instruments framework (COSMIN). Face validity was assessed through cognitive debriefing interviews (CDIs). Construct validity was assessed by testing 5 *a priori* hypotheses with the EQ-5D-5L instrument, using Spearman’s correlation coefficients. Test–retest reliability was evaluated using kappa, observed proportion of agreement and intraclass correlation coefficient (ICC), while the internal consistency was determined using Cronbach’s alpha.

**Results:**

Among 251 participants (mean age: 34.7, 83.6% females, 7.31 years of working experience), the NMQ-E demonstrated good internal consistency (Cronbach’s alpha above 0.81), and excellent test–retest reliability [ICC = 0.987 (95% CI 0.974–0.994)] with moderate to almost perfect reliability (kappa = 0.64–0.95). All *a priori* were confirmed in convergent validity assessment. Item response rates exceeded 97%, indicating good feasibility.

**Conclusion:**

The NMQ-E is a valid and reliable instrument for assessing work-related musculoskeletal symptoms among healthcare professionals in community hospitals within Singapore. Its application may greatly support epidemiological surveillance and inform occupational health interventions in Singapore’s healthcare setting.

## 1 Introduction

Musculoskeletal disorders (MSDs) represent a public health challenge of serious concern and poses a high economic burden (1). According to the World Health Organization, the term MSDs encompasses health problems in the musculoskeletal system (i.e., muscles, tendons, joint tissue nerves, fascia and ligaments) (2). A high risk for development of work-related MSD exists in the healthcare profession (3–5). The healthcare sector is consistently ranked among the top few sectors with the most work-related MSDs. Data from the Labour Force Survey in Europe highlights that the healthcare sector has the second highest rate of MSDs, following the agricultural sector (6). Within the healthcare sector, MSDs rank the highest among all the reported work-related health problems (7). The adverse effects of MSDs on healthcare professionals, organizations and society include injuries and disabilities, work absenteeism, lower productivity and satisfaction (8). For healthcare professionals, this often translates to lower capacity for quality patient care and safety.

To facilitate interventions that address this problem, reliable data collection as part of epidemiological investigations addressing work-related MSDs is essential (9). Patient-reported outcome measures (PROMS) are widely used to screen for and monitor work-related MSDs (10). Among these, the standardized Nordic Musculoskeletal Questionnaire (NMQ), developed by Kuorinka et al. (11) is one of the most extensively used instruments. It quantifies musculoskeletal pain and related activity limitations across nine anatomical regions through a standardized set of 3 core questions. Kuorinka et al. (11) reported the NMQ test–retest reliability and validity in relation to clinical history in occupational groups (safety engineers, office workers, railway workers and production workers). The authors concluded that the NMQ is suitable for investigating musculoskeletal symptoms and for monitoring the effectiveness of occupational health interventions. While other instruments such as the Cornell Musculoskeletal Discomfort Questionnaire, QuickDASH and Oswestry Disability Index can similarly assess symptom severity and functional impact, they are typically region-specific—targeting regions like the upper limbs or lower back—and are more commonly applied in focused clinical settings.

To address the need for more comprehensive data, Dawson et al. (9) introduced the extended Nordic Musculoskeletal Questionnaire (NMQ-E), an expanded version of the NMQ. The NMQ-E includes additional items capturing symptom duration, severity and healthcare utilization and work-related consequences, making it suitable for collecting richer datasets on the burden of work-related MSDs. It was initially administered to health sciences students of nursing in Australia and assessed to be reliable and reproducible in both self and interviewer administration. However, limitations of that study included the absence of content validity and a short 24-h test–retest interval. Given its simplicity, relevance to occupational health and suitability for large-scale administration, the NMQ-E has since been translated and culturally adapted into Persian (12), Turkish (13) and Hebrew (14), with outcomes across general and occupational cohorts, respectively. As outcome measures are heavily influenced by socio-cultural context, it is critical to measure the psychometric properties of the NMQ-E in the local context before its use (9).

In Singapore, studies evaluating work-related MSDs among healthcare professionals have primarily utilized the original NMQ (15, 16), but none using the NMQ-E. Furthermore, there are no validated tools specifically adapted for assessing work-related MSD in the local context. This represents an important gap, particularly in community hospital settings where healthcare professionals are frequently exposed to physically demanding tasks such as rehabilitation activities and patient handling. Therefore, this study aimed to evaluate the validity and reliability of the NMQ-E among healthcare professionals working in community hospitals. Establishing a validated instrument for this context will enable more accurate assessment of the prevalence and severity among healthcare professionals in Singapore and facilitate the development of targeted interventions.

## 2 Materials and methods

### 2.1 Study design

We conducted this study in two phases in accordance to the Consensus-based Standards for the selection of Health Measurement Instruments (COSMIN) framework (17, 18). The measurement properties evaluated included content and construct validity, internal consistency, test–retest reliability and measurement error. The first phase involved cognitive interviews to examine the face validity of the instrument, while the second phase comprised a cross-sectional validation study. Informed consent was obtained from all participants prior to study participation. Sing Health Centralized Institutional Review Board (CIRB) approved this study (reference 2023–2,650).

#### 2.1.1 Cognitive debriefing and face validity

Prior to the main validation study, ten participants from Sengkang Community Hospital were purposively sampled to comment on the relevance, comprehensibility, and comprehensiveness of the NMQ-E instrument. The sample was chosen to represent a range of professions, ages, genders, race and years of work experience. Cognitive interviews were conducted by one trained interviewer (PEG) using a structured interview guide and in accordance with the Cognitive Interviewing Reporting Framework (19). Both think aloud and verbal probing techniques were utilized (20). Participants self-administered the NMQ-E in the presence of the interviewer while verbalizing their thought processes to identify any potential difficulties in item comprehension and interpretation. After completion, the interviewer used verbal probing to assess if the participants found the NMQ-E easy to understand, relevant and comprehensive. Other factors for consideration included any missing, ambiguous or inappropriate items. Feasibility of the instrument was assessed by recording the time it took to complete the NMQ-E.

#### 2.1.2 Cross-sectional validation study

This study was conducted among healthcare professionals working in two community hospitals in Singapore. Community hospitals play a key role in the continuity of care between tertiary institutions and the community, enabling patients to receive inpatient medical services within a community-based setting. They typically provide two main categories of inpatient services: rehabilitative care, which supports functional recovery after acute illness or surgery, and subacute care, which manages patients with ongoing medical needs not requiring specialist-level acute care. Eligible healthcare professionals who were currently employed and actively working in either of the hospitals were invited to participate in the study through institutional mass emails containing a brief introduction of the study and a study information sheet. Participation was voluntary. The healthcare professionals recruited comprised physiotherapists, occupational therapists, and nurses across all ranks. These occupational groups were selected due to their direct involvement in physically demanding clinical tasks and their increased exposure to work-related musculoskeletal risk factors, making them particularly relevant for the objectives of this study. Additional inclusion criteria were: age 21 years old or older, and English-speaking. Participants were excluded if they worked less than 1 year in their current workplace, had severe chronic systemic or musculoskeletal diseases (e.g., inflammatory rhematic diseases, fibromyalgia, recent musculoskeletal trauma less than 6 months ago), or unable to provide informed consent. Healthcare professionals who were not actively working at the time of recruitment, such as those on prolonged leave of absence, were not eligible for participation. No personal identifiers were collected in order to preserve the anonymity of responses.

Eligible and interested participants were asked to complete a questionnaire comprising sociodemographic characteristics, the NMQ-E and EQ-5D-5L instruments. For a subset of participants, the NMQ-E was assessed at 2 weeks (day 14) after baseline assessment. The 14-day interval was selected as per recommendations by COSMIN framework, which allows for an adequate length to minimize recall effects while providing an indicator of non-specific score changes that occur naturally in a PROM instrument (21).

### 2.2 Instruments and definitions

#### 2.2.1 NMQ-E

The NMQ-E comprises 99 questions and can be self-administered or completed via face-to-face interview (9). It gathers information on the onset and prevalence of musculoskeletal pain in nine separate body regions (neck, shoulders, upper back, elbows, wrists/hands, lower back, hips/thighs, knees, and ankles/feet) and over four distinct time periods (lifetime, annual, monthly and present). Furthermore, the NMQ-E examines the impact of musculoskeletal discomfort encountered in the above-mentioned body regions on work and daily activities. Information on the need for medical care (e.g., hospitalization, medication and visiting healthcare providers) owing to said symptoms are also collected. Apart from age data, all response options are dichotomous (yes/no). Questions are arranged in the order such that those relating to the respondents’ lifetime (“ever”) were asked first, followed by prevalence questions, and lastly items relating to consequences of pain. Participants were asked to answer all questions about a body region before progressing to the next region (i.e., horizontally rather than vertically).

#### 2.2.2 EQ-5D-5L (EuroQOL-5 dimensions-5 levels questionnaire)

The EQ-5D-5L is a PROM measuring health-related quality of life. It comprises a health descriptive component and a visual analogue scale (VAS) (22). The descriptive component examines five items related to healthcare encompassing mobility, self-care, usual activities, pain/discomfort, and anxiety/depression. There are five possible responses for each item, ranging from ‘no problems’ to ‘extreme problems’. Scores from the five items can then be used to derive a composite score ranging from −1.00 to 1.00, indicating the worst possible health state to the perfect health state.

The VAS is a 20 cm vertical scale that is scored from 0 to 100 points. As with the health descriptive component, the 2 scores represent the “worst imaginable health state” and the “best imaginable health state” respectively. The EQ-5D-5L has been validated for use among patients with axial spondylarthritis in Singapore (23), and further information on the instrument can be found on the official EuroQoL website (24).

### 2.3 Statistical analysis

Data analyses were planned *a priori* and analyzed with STATA SE 18 for Windows (StataCorp, College Station, Texas). We excluded participants with missing data from the analysis. Categorical variables were expressed as frequency (n) and percentage (%), while continuous variables were reported as “mean ± SD.”

Pertaining to sample size computation, there are currently no guidelines on sample size computation for validation of the NMQ-E tool. However, it is generally recommended by the COSMIN guidelines to have at least 50–100 respondents (21).

### 2.4 Content validity

To analyze content validity, we invited 5 healthcare reviewers who are experts in their respective fields (family physician, occupational medicine specialist, nurse, occupational therapist and physiotherapist) to complete the questionnaire independently and rank the clarity and relevance of each item on a rating scale of 1 to 4, where 1 = unclear/irrelevant, 2 = somewhat unclear/irrelevant, 3 = clear/relevant and 4 = very clear/very relevant (20, 4). They were also asked to assess the questionnaire for relevance to the target population and construct of interest, if the response options were appropriate, and if key concepts were missing. The item-level content validity index (I-CVI) was calculated as the proportion of experts rating each item as either 3 or 4 (25, 26). The scale-level content validity index (S-CVI) was calculated as the average of the I-CVI values across all items. An acceptable cut-off value for I-CVI and S-CVI when evaluated by 5 experts is ≥0.78 and 0.9, respectively, (25, 26).

### 2.5 Construct validity

Validity refers to the extent which the tool can quantify what it seeks to measure. Convergent construct validity is a subset of construct validity which determines if items of the instrument are consistent with that of other related instruments through hypotheses testing (21). The construct of interest was musculoskeletal symptom burden, defined as the presence, distribution and functional impact of musculoskeletal symptoms across multiple anatomical regions. The NMQ-E is intended to measure this construct through self-reported symptoms and their consequences on work and daily activities. Construct validity between the NMQ-E sub-scales and that of EQ-5D-5L was evaluated using Spearman rank coefficients (*r_s)_*. High (*r_s_* = 0.5–0.8) and moderate correlation coefficients (*r_s_* = 0.3 < 0.5) indicate that the scores from the two PROMs are correlated, while low correlation coefficients (*r_s_* ≤ 0.3) indicate that the PROMs are quantifying different constructs (27). The validities of convergent constructs were evaluated with 5 *a priori* hypotheses based on established theoretical relationships between musculoskeletal symptom burden and health-related quality of life. Given that the 2 instruments assess overlapping but distinct domains, stronger correlations were anticipated where the constructs were closely aligned (e.g., pain/discomfort) while weaker correlations were hypothesized for broader domains (e.g., anxiety/depression), consistent with prior psychometric literature (28–30). Construct validities of the EQ-5D-5L were supported if at least 75% of the results are in accordance with hypotheses (31). A *p-*value of <0.01 was considered statistically significant after applying Bonferroni’s correction.

The hypotheses were as follow:

1. Sum of score for “Trouble in any body region today” in NMQ-E is positively and strongly correlated with higher scores in “Pain/discomfort” dimension in EQ-5D-5L questionnaire.
2. Sum of score for “Trouble in any body region today” in NMQ-E is positively and weakly correlated with higher scores in “Usual Activities” dimension in EQ-5D-5L questionnaire.
3. Sum of score for “Trouble in any body region today” in NMQ-E is negatively and moderately correlated with scores in EQ VAS score in EQ-5D-5L questionnaire.
4. Impact of trouble (questions on trouble preventing participant from doing normal work or having to see doctor or taking medication/MC in NMQ-E) positively and weakly correlated with higher scores in “Usual Activities” dimension EQ-5D-5L questionnaire.
5. Impact of trouble (questions on trouble preventing participant from doing normal work or having to see doctor or taking medication/MC in NMQ-E) positively and weakly correlated with higher scores in “Anxiety/Depression” dimension EQ-5D-5L questionnaire.

### 2.6 Reliability

Reliability refers to the overall consistency of an instrument. Observed values in a reliable instrument are true within acceptable errors of measurements (32). Reliability was studied using internal consistency, assuming all items were similar and measured a single construct. Internal consistency was estimated using Cronbach’s alpha and was supported only if it was more than 0.7 (33). Test–retest reliability was also investigated using Cohen’s unweighted kappa (*ĸ*) and the proportion of observed agreement (*P_o_*) for dichotomous variables in the NMQ-E and intraclass correlation coefficient (ICC) (two-way mixed effects model) with a 95% confidence interval (CI) for the continuous variables such as age. Kappa values of ≤ 0.2, 0.21–0.4, 0.41–0.6, 0.61–0.8 and ≥ 0.81 indicates poor, fair, moderate, good and very good agreement of the responses, respectively, between the repeated evaluations (34), while the proportion of agreement was interpreted as follows: <0, less than chance agreement; 0.01–0.2, slight agreement; 0.21–0.40, fair agreement; 0.41–0.6, moderate agreement; 0.61–0.8, substantial agreement; and more than 0.81 to be almost perfect agreement. Excellent reliability is demonstrated by an ICC that is greater than or equal to 0.7 (17). The standard error of measurement (SEM) was calculated using the following formulae: SEM = SD *x* √(1 –ICC) (SD is the standard deviation).

## 3 Results

### 3.1 Face validity with CDI

Of the 10 participants interviewed, 4 of them were nurses while the rest were occupational and physiotherapists. All of them had varying work experience and were of different levels of seniority. No item was skipped by the participants during self-administration. The average completion time was approximately 5 min (range: 4–7 min). All participants reported that the content in the instrument was relevant and easy to understand. Two participants required clarification for the meaning behind “change of duties” in item 4. Another 3 participants questioned if traditional Chinese medicine (TCM) practitioners or its equivalents were included in the reference “other such person” in item 10. The NMQ-E was then modified (Table 1) to include these clarifications after updating the developer and further tested in another 5 participants. Post-modification, there were no reported difficulties or ambiguity encountered. Participants also did not identify any missing domains, suggesting that the instrument adequately captured the construct of interest. This adapted instrument was therefore used in the cross-sectional study (Supplementary Additional File S1).

**Table 1:** Modifications made to the NMQ-E instrument.

Item	Original question	Modified question
4	Have you ever had to change jobs or duties (even temporarily) because of the trouble?	Have you ever had to change jobs or duties, e.g., *light duty* (even temporarily) because of the trouble?
10	During the last 12 months, have you at any time seen a doctor, physiotherapist, chiropractor or other such person because of the trouble?	During the last 12 months, have you at any time seen a doctor, physiotherapist, chiropractor, *TCM practitioner* or other such person because of the trouble?

NMQ-E, extended Nordic musculoskeletal questionnaire.

### 3.2 Cross-sectional validation study

A total of 251 participants from SKCH and OCH were recruited for our study from March 2024 to August 2024. No participants were excluded from our study. Among them, the mean age was 34.7 years old, with 7.31 years of working experience, and 36.87 h of direct patient contact per week (Table 2). Majority of them were female (*n* = 169, 83.7%). Only 6% of participants reported a pre-existing musculoskeletal disorder, and 8.7% reported a history of chronic disease, indicating that most participants did not have major known baseline comorbidities.

**Table 2:** Sociodemographic characteristics of participants in cross-validation study (*n* = 251).

Characteristics	Mean or *n* (%)
Age, mean (SD)	34.69 (8.61)
Gender, *n* (%)	
Male	33 (16.34)
Female	169 (83.66)
BMI, mean (SD)	24.06 (4.69)
Occupation, *n* (%)	
Nurses	149 (74.50)
Therapists	51 (25.5)
Years of working experience, mean (SD)	7.31 (6.05)
Hours of direct patient contact per week, mean (SD)	36.87 (10.47)
Amount of moderate-intensity exercise per week, mean (SD)	2.13 (4.25)

BMI, Body mass index; SD, Standard deviation.

Feasibility was calculated by the proportion of missing item responses, with high overall completion rates ≥ 90% taken as evidence of questionnaire acceptability. Response rate for all items exceeded 97%, indicating good feasibility of the instrument.

To better characterize the study sample, the distribution of self-reported musculoskeletal symptoms across body regions and recall periods was reported in Table 3. The low back was the most commonly affected region, with a lifetime prevalence of 70.3%, and a preceding annual and 1-month prevalence of 52.0 and 42.1%, respectively.

**Table 3:** Reported pain prevalence of the participants during different time periods (%).

	Lifetime pain	Pain in last 12 months	Pain in the last month	Pain today
Neck	50.1	35.2	27.4	14.5
Shoulders	59.4	43.1	34.3	18.5
Upper back	44.1	31.7	22.9	16.5
Elbows	15.8	7.9	8.4	5.0
Wrists	40.6	27.7	21.9	11.9
Low back	70.3	52.0	42.1	26.4
Hips/thighs	25.7	18.8	13.9	8.4
Knees	38.6	28.7	19.8	11.4
Ankles/feet	47.0	33.7	27.7	18.3

### 3.3 Content validity

All 5 expert reviewers endorsed the relevancy and validity of the instrument, resulting in an I-CVI and S-CVI of 1.00. No further modifications were required.

### 3.4 Convergent validity

Table 4 shows the convergent validity of the NMQ-E instrument. As hypothesized, the NMQ-E correlated strongly and was statistically significant with the pain/discomfort dimension in EQ-5D-5L (*r* = 0.52, *p* < 0.0001). The sub-scales of “trouble in any body region today” and “impact of trouble” of the NMQ-E correlated weakly with the EQ-5D-5L “usual activities” domain (*r* = 0.23 and 0.25, *p* = 0.002 and 0.0006). Construct validity of the NMQ-E was supported, as all 5 *a priori* hypotheses were confirmed in the results. This exceeded the COSMIN threshold of 75% for adequate construct validity.

**Table 4:** Construct validity of NMQ-E instrument.

Hypothesis	Magnitude	Direction	Actual correlation	Hypothesis met
“Pain/discomfort” dimension (EQ-5D-5L) with “Trouble in body region” (NMQ-E)	Strong	+	0.52	Yes
“Usual activities” dimension (EQ-5D-5L) with “Trouble in body region” (NMQ-E)	Weak	+	0.22	Yes
EQ -5D-5L VAS score with “Trouble in body region” (NMQ-E)	Moderate	−	0.42	Yes
“Usual activities” dimension (EQ-5D-5L) with “Impact of work” (NMQ-E)	Weak	+	0.25	Yes
“Anxiety/depression” dimension (EQ-5D-5L) with “Impact of work” (NMQ-E)	Weak	+	0.27	Yes

EQ 5D-5L, EuroQOL-5 dimensions-5 levels; NMQ-E, extended Nordic musculoskeletal questionnaire.

### 3.5 Internal consistency and test–retest reliability

The results of the internal consistency analysis are presented in Table 5. Cronbach’s alpha of the NMQ-E was consistently above 0.8, which demonstrated good internal consistency.

**Table 5:** Internal consistency and test–retest reliability of the NMQ-E: age of onset and prevalence questions.

	Age at the onset		Annual prevalence	Month prevalence	Point prevalence	Internal consistency^*^
	ICC (95% CI)	SEM	ĸ	ĸ	ĸ	
Neck	0.998 (0.995–0.999)	0.042	0.76	0.71	0.64	0.85
Shoulders	0.995 (0.991–0.998)	0.072	0.83	0.78	0.58	0.85
Upper back	0.997 (0.993–0.999)	0.048	0.79	0.83	0.53	0.87
Elbows	1	0.000	0.69	0.79	0.78	0.89
Wrists	0.999 (0.999–1.000)	0.008	0.64	0.64	0.56	0.88
Low back	0.987 (0.974–0.994)	0.139	0.91	1.00	0.65	0.81
Hips/thighs	0.999 (0.997–1.000)	0.019	0.84	0.83	0.57	0.86
Knees	0.997 (0.993–0.999)	0.042	0.95	1.00	0.85	0.84
Ankles/feet	0.999 (0.998–1.000)	0.010	0.91	0.91	0.84	0.87

CI, Confidence interval; ICC, Intraclass correlation coefficient; NMQ-E, Extended Nordic Musculoskeletal Questionnaire; SEM, Standard error of measurement; ĸ, Cohen’s unweighted kappa. *Included (i) ever had trouble, (ii) annual prevalence, (iii) month prevalence and (iv) point prevalence.

The test–retest reliability of the NMQ-E was evaluated in 50 participants, and the results are seen in Table 6. The ICC values for the age of onset of “trouble” question showed excellent test–retest reliability, with values ranging from 0.995 in the shoulder region, to 1 in the elbow region. Similarly, the reliability for monthly and annual prevalence across the different body regions was good, with a Cohen’s kappa correlation coefficient ranging between 0.64–0.95. However, the kappa coefficient was moderate in the ‘impact of trouble’ domain for the shoulders and low back regions. For body regions such as the elbows and wrists, it was not possible to compute the correlation coefficient test because the same and constant answers were given in both two conditions.

**Table 6:** Test–retest reliability of the NMQ-E with ĸ and *P_o_*: Questions on impact of pain.

	Lifetime hospitalization		Lifetime changed job		Trouble caused by job		Annual prevention of normal work		Annual visit to healthcare providers		Annual medication		Annual sick leave	
	ĸ	*P_o_*	ĸ	*P_o_*	ĸ	*P_o_*	ĸ	*P_o_*	ĸ	*P_o_*	ĸ	*P_o_*	ĸ	*P_o_*
Neck	1.00	0.98	0.66	0.96	0.71	0.86	0.65	0.94	0.64	0.92	0.80	0.92	0.85	0.96
Shoulders	1.00	0.98	0.66	0.96	0.75	0.86	0.55	0.90	0.83	0.94	0.75	0.88	0.85	0.96
Upper back	1.00	0.98	1.00	0.98	0.79	0.88	0.73	0.92	0.78	0.94	0.90	0.94	0.90	1.00
Elbows	1.00	0.98	1.00	0.98	0.83	0.96	*	0.96	0.66	0.96	0.48	0.94	1.00	1.00
Wrists	1.00	0.96	0.66	0.94	0.74	0.84	*	0.92	0.66	0.94	0.69	0.90	*	0.94
Low back	0.66	0.94	0.79	0.94	0.87	0.90	0.79	0.90	0.56	0.86	0.76	0.86	0.56	0.84
Hips/thighs	0.79	0.94	0.66	0.94	0.90	0.92	0.84	0.94	0.79	0.94	0.74	0.90	0.79	0.94
Knees	0.66	0.94	1.00	0.96	0.78	0.88	1.00	0.96	0.66	0.94	1.00	0.96	1.00	0.96
Ankles/feet	1.00	0.96	0.66	0.94	0.87	0.90	0.73	0.92	0.79	0.94	1.00	0.96	1.00	0.96

NMQ-E, Extended Nordic Musculoskeletal Questionnaire; ĸ, Cohen’s unweighted kappa; Po, proportion of observed agreement.

*Indicates that a metric cannot be calculated.

## 4 Discussion

Our study findings support the use of the NMQ-E as a valid and reliable tool in assessing work-related musculoskeletal symptom burden among healthcare professionals in Singapore’s community hospitals. To the best of our knowledge, this is the first study to evaluate the psychometric properties of the NMQ-E in a Singapore healthcare setting, addressing an important gap in locally validated occupational health measurement tools. A key strength of this study is its adherence to the COSMIN framework, including the formulation of *a priori* hypotheses for construct validation and the evaluation of multiple measurement properties.

Following our cognitive debriefing interviews, we modified the NMQ-E by refining definitions to two items in the instrument. Given Singapore’s diverse multi-cultural workforce and unique occupational context, the semantic interpretation, idiomatic understanding and health belief models can differ significantly from other countries. Traditional Chinese medicine and its equivalents are commonly used for managing musculoskeletal complaints in Singapore (35), and its inclusion in the healthcare-seeking item was necessary to reflect culturally relevant healthcare practices. Similarly, the term “light duty” was included as an example in the job modification item as it is the most common and recognizable form of temporary job change or restriction locally due to musculoskeletal conditions. Cross-cultural adaption using CDIs ensure that the psychometric integrity of the NMQ-E is preserved when used in our local context. Importantly, these modifications enhanced interpretability without altering the underlying constructs measured by the measurement, thereby preserving its psychometric integrity. Content validity was also evaluated by a panel of experts and deemed excellent through calculation of CVI. While the number of experts was modest, the high level of consensus regarding item relevance was consistent with participant feedback, further supporting the relevance and comprehensiveness of the instrument.

To evaluate construct validity, hypotheses testing was conducted as there is currently no instrument available which can evaluate the prevalence and severity of symptoms concurrently in all 9 body regions. Construct validity was supported with the confirmation of all hypothesized associations between the NMQ-E and EQ-5D-5L instruments, meeting the COSMIN criteria for sufficient construct validity. The observed pattern of correlations, with stronger associations in conceptually related domains and weaker associations in less related domains, is consistent with theoretical expectations and indicates that the NMQ-E adequately captures musculoskeletal symptom burden.

Overall, the reliability of the NMQ-E was demonstrated through evaluation of internal consistency and test–retest reliability. Using kappa statistics, the test–retest reliability of the NMQ-E for prevalence questions was high with the agreement index ranging between 0.81 to 1, except for point prevalence. This is similar to the original study and the various translated versions (9, 12, 14, 36). In comparison, the items on annual medication, sick leave and prevention of work displayed moderate agreement. Our results reflect similar trends to that of the original NMQ-E. As elucidated by Dawson et al., this is likely attributed to the presence of low prevalence. This leads to a higher proportion of agreement expected by chance, which translates to a low kappa during the chance correction process (9). Future studies should consider the use of additional indices such as the prevalence-adjusted bias-adjusted kappa, where prevalence and bias are accounted for in statistical calculations, potentially yielding a better agreement estimate (37). It is noteworthy that despite having a longer timeframe of 14 days for test–retest interval, our results are comparable to the existing studies which used time intervals of 24 h to 7 days, attesting to stability over time. While this reduced the likelihood of recollection bias, it increased the chance of point prevalence differences. Like previous studies, our study revealed an excellent ICC for the “age of onset” item, further proving its reliability (13, 14, 36). Moreover, the low SEM scores reported in our study indicates that the NMQ-E has high absolute reliability.

Pertaining to internal consistency, Cronbach’s alpha for our study exceeded 0.8, indicating good internal consistency across body region subscales of the NMQ-E. While Dawson et al. did not examine internal consistency for the original NMQ-E, other studies reported alpha values ranging from 0.78–0.95, which were concordant with our findings (12, 13, 38). This supports the reliability of the instrument in our population of interest.

### 4.1 Implications

The findings of our study have important implications for both clinical and research practice. As the first study to evaluate the measurement properties of the NMQ-E among healthcare professionals in Singapore’s community hospitals, it addresses a significant gap in the availability of locally validated instruments which can support standardized assessment of musculoskeletal symptoms and their impact. In practice, the NMQ-E may be useful for screening, documentation and surveillance of musculoskeletal symptom burden across relevant occupational groups. Its structured and convenient format allows systematic identification of symptom distribution across body regions. The information gleaned can subsequently be used to assist healthcare institutions in identifying symptom patterns, affected body regions and areas where further evaluation may be indicated. It is noteworthy to highlight that the NMQ-E should not be used in isolation for clinical decision-making or planning interventions. Given that the NMQ-E is a self-reported questionnaire, its results will be most useful when combined with clinical assessment, ergonomic evaluation and qualitative interviews with employees regarding task demands, coping strategies and barriers to sustained work participation. This contributes to a more comprehensive understanding of work-related musculoskeletal health and can help inform workplace support strategies.

From a research perspective, the availability of a locally adapted and validated version of the NMQ in the Singapore context may facilitate consistent data collection in future epidemiological, occupational health and implementation studies. This allows for reliable longitudinal tracking of workplace intervention efficacy and identification of systemic risk factors across the different healthcare clusters.

### 4.2 Limitations

Several limitations should be considered. First, it focused on a specific subset of healthcare professionals, namely nurses, occupational therapists and physiotherapists. While this approach ensured more accurate content and construct validation of the NMQ-E by ensuring alignment with similar job roles and physical task profiles, it may limit the generalizability of the findings to other occupational populations. Second, while the dichotomous nature of the response options in the NMQ-E facilitates ease of completion, it may reduce sensitivity to graduations in symptom severity, fluctuation and context. This limits its use as a stand-alone tool for planning individualized interventions to improve work participation. Third, the reproducibility of online administration methods was not explored in this study. In addition, the absence of a recognized gold standard comparator for musculoskeletal symptom assessment precluded the evaluation of criterion validity. Finally, while our study adhered to COSMIN framework, certain measurement properties—such as responsiveness—could not be assessed due to the cross-sectional study design. Nevertheless, our study serves as a foundation for future psychometric evaluation in distinct occupational cohorts and in longitudinal research contexts.

### 4.3 Future directions

Future research should consider conducting cross-validation in a broader range of healthcare and non-healthcare occupations to strengthen applicability across diverse work settings and support its use as a national benchmarking tool for occupational health in Singapore. Furthermore, given Singapore’s focus on digital health initiatives, the psychometric properties of the NMQ-E within digital platforms can be explored. This would facilitate large-scale musculoskeletal surveillance, which allows for early identification of “pain clusters” and just-in-time ergonomic interventions. Longitudinal study designs will also be important to determine the NMQ-E’s responsiveness to change, particularly in assessing longitudinal efficacy of workplace health programs or clinical rehabilitation.

## 5 Conclusion

This study demonstrates that the NMQ-E has good content and construct validity, internal consistency and test–retest reliability among healthcare professionals working in Singapore community hospitals. As the first local validation of the instrument in this occupational setting, the results of this study provide a vital foundation for the standardized assessment of musculoskeletal symptom burden in the region. While the NMQ-E is an effective initial screen for identifying employees with musculoskeletal symptoms, its utility is enhanced through the addition of objective clinical assessment and qualitative feedback. Further research should delve into the responsiveness of the NMQ-E and its role within more comprehensive occupational health assessment frameworks.

## Data Availability

The raw data supporting the conclusions of this article will be made available by the authors, without undue reservation.
