# Supplementary material for: Validation of the extended Nordic Musculoskeletal Questionnaire among healthcare professionals in Singapore
Source: Front Public Health. 2026 May 19;14:1840201. doi: 10.3389/fpubh.2026.1840201 (PMC13226543; doi:10.3389/fpubh.2026.1840201)
Supplement: Supplementary file 1 [file Data_Sheet_1.pdf]

Additional File 1: Extended Musculoskeletal Questionnaire

Instructions:

Please answer by putting a cross in the appropriate box - one cross for each question. ***Answer every question, even if you have never had trouble in any part of your body. Please answer questions from left to right before going down to the next body region.*** This picture shows how the body has been divided. Limits are not sharply defined and certain parts overlap. You should decide for yourself which part (if any) is or has been affected.

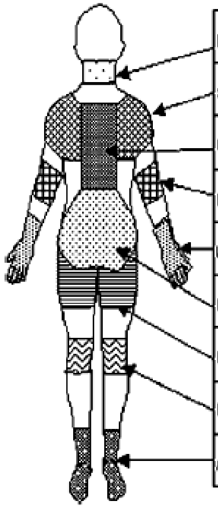

	Have you ever had trouble (ache, pain or discomfort) in:		At the time of initial onset of the trouble, what was your age?	Have you **ever** been hospitalised because of the trouble?	Have you ever had to change jobs or duties e.g., light duty (even temporarily) because of the trouble?	Have you had trouble (ache, pain, discomfort) at any time during the **last 12 months?**		Have you had trouble (ache, pain, discomfort) at any time during the **last month (4 weeks)?**	Have you had trouble (ache, pain, discomfort) **today?**	Do you think your trouble (ache/pain/ discomfort) is caused by work or aggravated by your work?	During the **last 12 months** have you at any time:			
											been prevented from doing your normal work (at home or away from home) because of the trouble?	Seen a doctor, physio-therapist, chiropractor, TCM practitioner or other such person because of the trouble?	taken medication because of the trouble?	taken sick leave from work/ studies because of the trouble?
NECK	☐ No ☐ Yes	***If 'No', go on to the next body region. If 'yes', please continue***	___ years	☐ No ☐ Yes	☐ No ☐ Yes	☐ No ☐ Yes	***If 'No', go on to the next body region. If 'yes', please continue***	☐ No ☐ Yes	☐ No ☐ Yes	☐ No ☐ Yes	☐ No ☐ Yes	☐ No ☐ Yes	☐ No ☐ Yes	☐ No ☐ Yes
SHOULDERS	☐ No ☐ Yes		___ years	☐ No ☐ Yes	☐ No ☐ Yes	☐ No ☐ Yes		☐ No ☐ Yes	☐ No ☐ Yes	☐ No ☐ Yes	☐ No ☐ Yes	☐ No ☐ Yes	☐ No ☐ Yes	☐ No ☐ Yes
UPPER BACK	☐ No ☐ Yes		___ years	☐ No ☐ Yes	☐ No ☐ Yes	☐ No ☐ Yes		☐ No ☐ Yes	☐ No ☐ Yes	☐ No ☐ Yes	☐ No ☐ Yes	☐ No ☐ Yes	☐ No ☐ Yes	☐ No ☐ Yes
ELBOWS	☐ No ☐ Yes		___ years	☐ No ☐ Yes	☐ No ☐ Yes	☐ No ☐ Yes		☐ No ☐ Yes	☐ No ☐ Yes	☐ No ☐ Yes	☐ No ☐ Yes	☐ No ☐ Yes	☐ No ☐ Yes	☐ No ☐ Yes
WRISTS/ HANDS	☐ No ☐ Yes		___ years	☐ No ☐ Yes	☐ No ☐ Yes	☐ No ☐ Yes		☐ No ☐ Yes	☐ No ☐ Yes	☐ No ☐ Yes	☐ No ☐ Yes	☐ No ☐ Yes	☐ No ☐ Yes	☐ No ☐ Yes
LOW BACK	☐ No ☐ Yes		___ years	☐ No ☐ Yes	☐ No ☐ Yes	☐ No ☐ Yes		☐ No ☐ Yes	☐ No ☐ Yes	☐ No ☐ Yes	☐ No ☐ Yes	☐ No ☐ Yes	☐ No ☐ Yes	☐ No ☐ Yes
HIPS/ THIGHS	☐ No ☐ Yes		___ years	☐ No ☐ Yes	☐ No ☐ Yes	☐ No ☐ Yes		☐ No ☐ Yes	☐ No ☐ Yes	☐ No ☐ Yes	☐ No ☐ Yes	☐ No ☐ Yes	☐ No ☐ Yes	☐ No ☐ Yes
KNEES	☐ No ☐ Yes		___ years	☐ No ☐ Yes	☐ No ☐ Yes	☐ No ☐ Yes		☐ No ☐ Yes	☐ No ☐ Yes	☐ No ☐ Yes	☐ No ☐ Yes	☐ No ☐ Yes	☐ No ☐ Yes	☐ No ☐ Yes
ANKLES/ FEET	☐ No ☐ Yes		___ years	☐ No ☐ Yes	☐ No ☐ Yes	☐ No ☐ Yes		☐ No ☐ Yes	☐ No ☐ Yes	☐ No ☐ Yes	☐ No ☐ Yes	☐ No ☐ Yes	☐ No ☐ Yes	☐ No ☐ Yes
